# The Nigerian juvenile justice system: from warehouse to uncertain quest for appropriate youth mental health service model – RETRACTION

**DOI:** 10.1192/bji.2018.18

**Published:** 2022-05

**Authors:** O. Atilola, G. Abiri, B. Ola

As a result of a technical error at the Publisher, the same article was erroneously published twice, once on the 19^th^ of March, 2018 (https://doi.org/10.1192/bji.2017.37), and again on the 23^rd^ of April, 2018. Subsequently it is necessary to retract the second appearance of the article in favour of the first for there to be a single version of record, though there is no issue with the article itself. Cambridge University Press sincerely apologise to the authors and readers for this mistake.
